# Beliefs About Pregnancy and Perceptions of Clinical Care Influencing the Uptake of Antenatal Care and Facility-Based Delivery: Findings Across the CHAMPS Network

**DOI:** 10.4269/ajtmh.26-0519

**Published:** 2026-11-10

**Authors:** John Blevins, Maria Maixenchs, Annie Hardison-Moody, Dalia Yeasmin, Farhana Hasnat Khan, Syead Tamim Mammud, Emily S. Gurley, Shahana Parveen, Constantino Cumbane, Quique Bassat, Helena Boene, John Anthony Gerard Scott, Nega Assefa, Lola Madrid, Getahun Wakwaya Sori, Wahibo Mohammed, Peter Otieno, Kelvin Akoth, Josephine Ouma, Kennedy Ochola, Hellen Muttai, Abdulai Othman Bah, Ikechukwu Udo Ogbuanu, Salieu Sesay, Martin Seppeh, Portia Mutevedzi, Kristin LaHatte, Solveig Cunningham, Chris A. Rees, Chelsea Cole, Jonathan A. Muir, Fauzia A. Malik, Montana Bartole, Mekleet Faltamo, El-Frida Pambo, Lyvannak Sam, Sophie Clarke, Cynthia G. Whitney

**Affiliations:** ^1^ Rollins School of Public Health, Hubert Department of Global Health, Emory University, Atlanta, USA;; ^2^ ISGlobal, Barcelona, Spain;; ^3^ North Carolina State University, Raleigh, North Carolina, USA;; ^4^ International Centre for Diarrhoeal Disease Research, Bangladesh (icddr,b), Dhaka, Bangladesh;; ^5^ Bloomberg School of Public Health, Johns Hopkins University, Baltimore, Maryland, USA;; ^6^ Centro de Investigação em Saúde de Manhiça (CISM), Maputo, Mozambique;; ^7^ Catalan Institution for Research and Advanced Studies (ICREA), Barcelona, Spain;; ^8^ Institut Clínic de Medicina I Dermatologia, Hospital Clínic de Barcelona, Barcelona, Spain;; ^9^ Facultat de Medicina i Ciències de la Salut, Universitat de Barcelona, Barcelona, Spain;; ^10^ Pediatrics Department, Hospital Sant Joan de Déu, Universitat de Barcelona, Barcelona, Spain;; ^11^ CIBER de Epidemiología y Salud Pública, Instituto de Salud Carlos III, Madrid, Spain;; ^12^ Department of Infectious Disease Epidemiology, London School of Hygiene and Tropical Medicine, London, United Kingdom;; ^13^ Haramaya University, Harar, Ethiopia;; ^14^ Kenya Medical Research Institute, Kisumu, Kenya;; ^15^ FOCUS 1000, Freetown, Sierra Leone;; ^16^ Africa Research Collaborative, Freetown, Sierra Leone;; ^17^ Ernest Bai Koroma University of Science and Technology, Magburaka, Sierra Leone;; ^18^ The Taskforce for Global Health, Decatur, Georgia, USA;; ^19^ Division of Pediatric Emergency Medicine, School of Medicine, Emory University, Atlanta, Georgia, USA;; ^20^ Children’s Healthcare of Atlanta, Atlanta, Georgia, USA;; ^21^ UT Southwestern Medical Center, Dallas, Texas, USA

## Abstract

Although quality antenatal care (ANC) and facility-based delivery reduce maternal and under-5 mortality, various factors impede uptake of these services. We conducted a qualitative study to assess the influences of common perceptions and practices about pregnancy and birth outcomes on 1) understandings of the causes of complicated pregnancies and birth outcomes and 2) attitudes toward pregnancy care. The study consisted of semistructured interviews or focus group discussions with women currently pregnant for the first time; women who had delivered a healthy child to term; women who had experienced a miscarriage or stillbirth; clinical health providers; traditional health practitioners; religious leaders; husbands of pregnant women; and parents and in-laws of pregnant women. A total of 107 events (97 interviews and 10 focus group discussions) were conducted within the catchment communities of sites of the Child Health and Mortality Prevention Surveillance Network in Bangladesh, Ethiopia, Kenya, Mozambique, and Sierra Leone. Analyzing narrative data, we identified three topics relevant for describing beliefs and attitudes toward pregnancy care: 1) influences of clinical frameworks for understanding pregnancy, 2) influences of cultural frameworks for understanding pregnancy, and 3) specific alignment/tension between community members’ beliefs and pregnancy care. We concluded by highlighting two barriers: 1) suspicion between clinical and traditional providers and 2) the impact of cultural belief on the uptake of ANC, especially in the first trimester, and two recommendations: 1) identify trusted partners to facilitate collaboration and 2) encourage the expansion of low-cost pregnancy support services to improve uptake of ANC and facility-based delivery.

## INTRODUCTION

Antenatal care (ANC) and facility-based delivery (FBD) improve maternal and newborn health outcomes. The impact of inadequate ANC on neonatal mortality in low- and middle-income countries is significant.^1^^,^^2^ One 2017 study estimated a 55% reduction in neonatal mortality if pregnant women followed the WHO recommendations to have their first ANC visit within the first trimester and had at least four follow-up visits, with a 32% reduction if just one of those recommendations were met.^3^ A second study examining ANC impact on neonatal mortality in sub-Saharan African countries found a 39% reduction in mortality with at least one ANC visit.^4^

Evidence also suggests that ANC and FBD are associated with improved outcomes for mothers and children, although estimates of their effects vary across settings. One study in in Ethiopia linked a lack of ANC to increased risk of maternal death, and another in Zambia associated completion of recommended ANC visits with substantially lower odds of maternal death.^5^^,^^6^ FBD has similarly been associated with lower perinatal mortality. Although FBD has generally been associated with lower neonatal mortality, its relationship with maternal mortality is more complex because women experiencing serious obstetric complications are disproportionately referred to facilities, sometimes after delays in receiving care, which can result in higher observed maternal mortality among facility deliveries and complicate estimates of the protective effect of facility-based care.^7^ In addition, access to and use of ANC and FBD are also shaped by intersecting social and cultural factors, including beliefs about pregnancy and childbirth, gendered norms regarding decision-making, socioeconomic status, costs, and the availability and quality of services.^8^^,^^9^ Attention to these factors is important to ensuring that the benefits of ANC and FBD are distributed equitably across populations.^9^

As a longitudinal pregnancy and mortality surveillance program designed to identify the causes of maternal mortality and mortality under 5 years of age, the Child Health and Mortality Prevention Surveillance (CHAMPS) network supports efforts to increase use of ANC and FBD. CHAMPS pregnancy surveillance activities link women to ANC and track visits and outcomes, including the proportion of deliveries in facilities.^10^ As CHAMPS sites in Bangladesh, Ethiopia, Kenya, Mozambique, Nigeria, Pakistan, Sierra Leone, and South Africa began to implement pregnancy surveillance, each site’s social-behavioral science (SBS) teams conducted formative research to understand women’s perceptions of and beliefs about ANC, what could improve participation in CHAMPS’ pregnancy surveillance, and how women could subsequently be linked to ANC. Those SBS formative studies revealed a complex picture that reflected the influences of social–cultural factors found in other studies^9^ and suggested that any efforts to increase uptake of ANC and FBD might be strengthened by attending to these factors. For example, the CHAMPS South Africa site found that both psychosocial factors and personal experiences contributed to women’s ambivalence about ANC.^11^ Such factors were common across other sites as well, with many participants stating that enrollment in ANC would be a disincentive to participating in pregnancy surveillance.

Findings from these studies led some sites in the CHAMPS network to launch a coordinated, multisite qualitative study to understand knowledge and beliefs about pregnancy, attitudes and perceptions of pregnancy-related clinical care (ANC, obstetric, postpartum), and health-seeking behaviors informed by such beliefs and perceptions. The study aimed to understand the influences of common perceptions, beliefs, and practices related to pregnancy and birth outcomes on 1) individual and community understandings of the causes of complicated or uncomplicated pregnancies and birth outcomes and 2) attitudes toward antenatal, obstetric, and postpartum clinical care.

## MATERIALS AND METHODS

This qualitative study was guided by four research questions:

1. What cultural assumptions about pregnancy inform the meanings, perceptions, beliefs, and practices of women and their families and of the broader community regarding pregnancy, birth outcomes, and the health of newborns?
2. What are the barriers and facilitators that pregnant women experience in trying to access antenatal, obstetric, and/or postpartum health care services?
3. What cultural assumptions about pregnancy inform the meanings, perceptions, beliefs, and practices of clinical and traditional health care workers regarding pregnancy, birth outcomes, and the health of newborns?
4. What are the barriers and facilitators that clinical and traditional health care workers experience in providing antenatal, obstetric, and postpartum health care services?

### Study design.

This was a cross-site study using a phenomenological approach to understand complex human experiences^12^^,^^13^ such as pregnancy and how related beliefs and behaviors surrounding pregnancy influence uptake of pregnancy-related clinical care. Qualitative narrative data were obtained through key informant interviews and focus group discussions and managed through Dedoose (Sociocultural Research Consultants, LLC; Manhattan Beach, CA, USA), a desktop web-based application created for qualitative thematic data analysis.

### Study sites and population.

The study was conducted in five CHAMPS sites: Bangladesh, Ethiopia, Kenya, Mozambique, and Sierra Leone. Participants were recruited to participate in an individual key informant interview (KII) or a focus group discussion (FGD). A combination of purposive and snowball sampling techniques was used to identify potential participants to reflect diverse views, interests, priorities, and expectations. Table 1 summarizes the populations and sampling frames used.

**Table 1:** Sampling frame for the study

Pregnant Women	
Pregnant Women or Women Who Gave Birth in Last Year (first pregnancy)	2 KIIs
Pregnant Women or Women Who Gave Birth in Last Year (not first pregnancy)	1 FGD^†^ or 2 KIIs
Women Who Experienced a Miscarriage or Stillbirth	3 KIIs (at least one of each)
Husbands of Pregnant Women	1 FGD* or 2 KIIs
Parents and In-Laws of Pregnant Women	4 KIIs (2 couples)
Key Community Leaders	
Medical Providers; Nurses, Midwives, Community Health Workers	1 FGD* or 2 KIIs
Traditional Practitioners: Traditional Healers, Traditional Birth Attendants, Elder Women	1 FGD* or 2 KIIs
Religious Leaders (Traditions Reflecting Site Demographics)	3 KIIs
Other Community Leaders (Dependent on Context)	TBD by site^†^

FGD = focus group discussion; KII = individual key informant; TBD = to be determined.

* If convening FGDs for this group was not feasible, sites could conduct 2 KIIs in place of an FGD.

^†^ Each site may identify other community leaders whose perspectives are important (for example, a local elder or a village chief). Sites should determine number of additional interviews, up to two.

Sites were encouraged to conduct FGDs for target participants who were likely to share similar perspectives in which a group conversation may generate richer data; if such FGDs were not feasible, sites could opt to conduct at least two KIIs for that population instead. Where there were diverse perspectives among religious contexts or leaders, or where researchers felt a focus group setting might inhibit conversation around sensitive topics (e.g., women who experienced a stillbirth or miscarriage), KIIs were conducted. As a result of these considerations, far more KIIs (97) were conducted as opposed to FGDs (10). The interviews and focus groups used semistructured guides to ensure that the same topics were covered across similar events in an individual site and across sites, while also offering flexibility to probe distinct topics raised by participants. A different guide was developed for each group in the sampling frame. Each guide explored the same core topics of pregnancy, ANC, birth and labor, and postpartum care to align with the research questions. However, the wording of the questions and follow-up probes reflected the varied context of the study participants. Consistent with phenomenological studies in general, we developed the interview guides to focus on participants’ lived experience and concrete practices related to pregnancy before probing on the beliefs, meanings, or perceptions that informed such behaviors.^12^^,^^13^ Demographic information was collected for each participant, specifically age, gender, sampling frame representation, religion, education, number and ages of any minor children living in their household, employment, place of residence, and prior participation in other CHAMPS activities.

### Data collection and data management.

Key informant interviews and focus group discussions were conducted between June and December 2024. These events were digitally recorded and transcribed into English. If the event was conducted in English, SBS staff created a verbatim transcript; if an event was conducted in a local language, they first translated the recording into English before transcribing. Events conducted in Mozambique were translated and transcribed into Portuguese because of the greater fluency of the local SBS staff in that language. A study coordinator fluent in Portuguese conducted quality control on the coding of the manuscripts in Portuguese; English translations of excerpts and relevant quotes from the Mozambique site were provided by this study coordinator and the SBS lead in Mozambique, both of whom are fluent in both English and Portuguese. A total of 107 transcripts were uploaded into a shared password-protected project file on Dedoose, and demographic variables were linked to each transcript.

#### Data analysis.

Data analysis consisted of an initial round of rough coding. An initial coding tree based directly on the topics explored in the interview guides was uploaded into Dedoose. This process allowed each site to develop and link new codes into the existing codes in the coding tree to reflect the specific topics discussed in interviews and focus groups and still provide a framework for combining similar codes articulated by researchers across the five sites or for linking rough codes to the research questions once a second round of fine coding was carried out. During fine coding, we grouped excerpts according to five emergent, cross-topical themes: 1) the influences of clinical frameworks for understanding of pregnancy, 2) the influences of cultural frameworks for understanding pregnancy, 3) mothers’ health behaviors during pregnancy, 4) experiences of miscarriage or stillbirth, and 5) influences of psychosocial factors. This grouping process generated a second fine coding tree with all excerpts assigned as a subcode to one of these five themes. Rough coding was conducted by researchers at each site, whereas fine coding reflected consensus by two or more researchers working collaboratively between the sites and by SBS workstream leads in the Program Office of the CHAMPS network.

### Theoretical framing of culture.

A significant body of scholarship at the intersection of the social sciences and health sciences has demonstrated the complex influences of social–cultural frameworks on health beliefs and behaviors.^14^^–^^22^ Such frameworks often function preconsciously, functioning as a worldview. They inform our understanding of the world without our conscious awareness of such functions because we internalize them as a taken-for-granted way of seeing the world. This study employs the term “healthworld” from Germond and Cochrane, two scholars who have contributed to this field, to describe such worldviews,^14^ in our analysis of findings. Germond and Cochrane demonstrate the influence not only of clinical/medical frameworks but also of spiritual/religious ones for informing health beliefs across diverse societies, especially those outside of North America and Europe. We borrow their healthworlds concept because our research also revealed such influences.

### Findings.

We completed 107 events (97 interviews and 10 focus group discussions). Table 2 summarizes the number and types of interviews and FGDs completed by each of the five sites.

**Table 2:** Number of completed interviews or focus group by site

Type of Study Participant	Countries				
	Bangladesh	Ethiopia	Kenya	Mozambique	Sierra Leone
Pregnant Women or Women Who Gave Birth in Last Year (first pregnancy) Sampling Target (per site): 2 KIIs	2 KIIs	2 KIIs	2 KIIs	2 KIIs	2 KIIs
Pregnant Women or Women Who Gave Birth In Last Year (not first) Sampling Target (per site): 1 FGD or 2 KIIs	2 KIIs	4 KIIs	2 KIIs	2 KIIs	2 KIIs, 1 FGD
Women Who Experienced a Miscarriage or Stillbirth. Sampling Target (per site): 3 KIIs (at least one of each)	3 KIIs	3 KIIs	3 KIIs	5 KIIs	3 KIIs
Husbands of Pregnant Women Sampling Target (per site): 1 FGD or 2 KIIs	2 KIIs	1 FGD	1 FGD	1 KII	1 KII, 1 FGD
Parents and In-Laws of Pregnant Women Sampling Target (per site): 4 KIIs (2 couples)	4 KIIs	3 KIIs (with couples)	4 KIIs	4 KIIs	4 KIIs
Medical Providers; Nurses, Midwives, Community Health Workers Sampling Target (per site): 1 FGD or 2 KIIs	1 FGD	3 KIIs, 1 FGD	2 KIIs	1 FGD	3 KIIs, 1 FGD
Traditional Practitioners, Traditional Birth Attendants, Elder Women Sampling Target (per site): 1 FGD or 2 KIIs	1 FGD	4 KIIs	2 KIIs	2 KIIs	2 KIIs, 1 FGD
Religious Leaders (Traditions Reflecting Site Demographics) Sampling Target (per site): 3 KIIs	3 KIIs	3 KIIs	4 KIIs	3 KIIs	3 KIIs
Other Community Leaders (dependent on context) Sampling Target (per site): TBD by Site	1 KII	0	0	0	0
Total Number of Events (107 events across all sites)	17 KIIs; 2 FGDs	22 KIIs; 2 FGDs	19 KIIs; 1 FGD	19 KIIs; 1 FGD	20 KIIs; 4 FGDs

FGD = focus group discussion; KII = individual key informant; TBD = to be determined.

The larger pregnancy perceptions study generated five themes exploring a wide variety of topics. We limit our discussion of findings to three general topics. Two topics directly examine two of the five themes generated by the larger study that are relevant for understanding the influence of cultural beliefs about pregnancy and perceptions of clinical care on the uptake of ANC and FBD: 1) the influences of clinical frameworks for understanding pregnancy and 2) the influences of cultural frameworks for understanding pregnancy. The third topic describes individuals’ or community perceptions of ANC or FBD by analyzing participants’ responses that specifically addressed or described such perceptions. Although the relevant findings from the qualitative data are summarized below, the Supplemental Materials include an Excel file with all coded excerpts of text (redacted to protect participant confidentiality) from KII and FGD transcripts organized under these topics.

### Influences of clinical frameworks for understanding pregnancy.

#### Knowledge of complications.

Study participants had a broad and diverse knowledge of complications that can occur during pregnancy and childbirth and were aware of clinical responses to those complications. We defined this knowledge as a clinical framework used by participants. Such understandings influence perceptions of ANC, generally in positive ways. Although this is not surprising given participants’ experiences in accessing clinical care, the awareness of clinical frameworks for understanding pregnancy among participants with no direct clinical experiences was nonetheless substantial. Ninety-seven of 107 KIIs or FGDs were conducted with participants who were not clinical providers. Still, they named 295 clinical complications that can arise during pregnancy and delivery; grouping duplicates yielded 72 distinct responses. Table 3 shows the 15 complications with at least five responses, ordered by frequency.

**Table 3:** Descriptions of complications that women face during pregnancy or factors that contributed to clinical complications named by community members (nonclinicians)

Complications		
Cause of Miscarriage/Stillbirth (45)	Emotional Impact (25)	Abdominal Pain (15)
Nausea (13)	blood pressure (9)	multiple complications (9)
Breech Position (8)	back pain (6)	caesarean delivery (6)
Dizziness (6)	pain (6)	delay in presenting for care (5)
Mistrust (6)	sleep loss/sleep patterns (5)	problems urinating (5)

#### Experiences of clinical care.

Beliefs about clinical care were also influenced by participants’ own experiences or by commonly held attitudes in the community. When asked to describe the quality of clinical care for pregnancy that they directly received or their perception about the quality of such care offered in their community, 88.1% of responses (223 of 253) from participants who were not health care providers were coded as “good quality care.” Despite this initial positive response, follow-on probes revealed a more complex picture. Examples of poor clinical care were offered by 31 of 70 (44%) participants. Of those participants, 12 (39%) gave only examples of poor care in the follow-on probe, whereas 19 (61%) offered examples of both poor and good care. Descriptions of poor clinical care fell into two categories: providers’ unprofessional actions and direct experiences of poor care.

### Providers’ unprofessional actions.

Participants offered 13 examples of unprofessional behavior by the clinicians who saw them. Seven participants described dismissive or disrespectful attitudes from providers. A Kenyan woman talked about her experience with a miscarriage at 7 months of pregnancy. While returning home from a family event, this mother went into premature labor. She tried to access services at three different medical facilities. She was turned away from two facilities for inability to pay. At the third she was accused of trying to cause her own pregnancy loss and was denied emergency care for not having her ANC card. She returned home to retrieve the card and delivered the baby there prematurely before she could return to the facility. The baby was dead when she returned to the facility. Husbands participating in focus groups in Sierra Leone and Bangladesh reported harsh treatment of their wives and inattentive staff, and several comments from Kenya, Sierra Leone, and Bangladesh conveyed treatment delays and refusals until payment was provided; one participant reported theft of her belongings by facility staff.

### Experience of poor clinical care.

Participants interviewed across the five countries identified 18 examples of poor clinical care arising from the limitations of the clinical and community health systems in their communities. One woman in Bangladesh who experienced a miscarriage criticized the lack of patient education and communication she received, noting that she never received information about the medications she was prescribed and had to research them on her own. Various participants complained of the poor physical upkeep of medical facilities, with three noting that government facilities were less sanitary, less well-stocked, and less well-staffed. Another woman from Bangladesh noted that health assessments occurred over the phone but did not lead to in-person clinical care. A woman from Ethiopia questioned the value of being diagnosed with anemia when the provider had no medication or resources to treat it. She also noted that the suggested modifications to her diet were unrealistic. Participants from Ethiopia, Kenya, Mozambique, and Sierra Leone noted the lack of support provided to new mothers after delivery, stating that any support came from community networks and not clinical staff.

Twelve participants from all five countries described how long wait times and facilities lacking basic resources discouraged women from prioritizing ANC. The mother-in-law of a pregnant woman in Mozambique criticized facilities for their failure to offer even the most basic support to patients. A mother from Bangladesh described a delay in receiving oxygen that placed her fetus at risk. A mother in Ethiopia wondered why a life-changing diagnosis was not made until late in her pregnancy, even though she had attended ANC. Eight of these twelve participants referenced long travel distances as a barrier to care.

Clinical providers also complained of poor clinical capacities. One provider in Bangladesh noted long-standing gaps in clinical services. A provider in Ethiopia felt confident that clinical staff had the knowledge and skills to address complications but lacked the resources to effectively manage them.

#### Resources and services to improve clinical care.

Families who had recently experienced pregnancy were asked to suggest the most helpful resources and support that they wish they had received, offering insights on ways that providers can better support their patients. They made few references to highly technical clinical resources but focused on essential needs and respectful interactions between providers and patients. Suggested services included enhanced health education for fathers (6), more extensive health monitoring (1), and ultrasounds (1). Suggested resources included medication (4), diapers (3), mosquito nets (3), a hat for the infant (3), blankets (2), financial support (1), food (1), vitamins (1), and a wash basin.

All the responses above reflect an awareness of clinical care during pregnancy. Clinical services, however, are not the only elements that inform participants’ understandings of pregnancy. Women in local communities across the CHAMPS network are also influenced by cultural frameworks that provide information about pregnancy, the causes of pregnancy complications, and the rituals and acts to prevent those complications or address them if they occur. In short, clinical frameworks have influenced preexisting cultural beliefs and practices, but the clinical has not supplanted the cultural.

### Influences of cultural frameworks for understanding pregnancy.

Participants described the advice that women receive to ensure a healthy pregnancy; at times, such advice was contradictory. This advice included staying indoors at dusk, staying indoors at midday, staying indoors during the rain, avoiding a visit to a home where a death has occurred, avoiding more strenuous household tasks, maintaining strenuous household tasks to ensure a healthy baby, eating a healthier diet with more calories to support fetal health, minimizing caloric intake to discourage the baby from growing too large and complicating delivery, binding the womb to protect the fetus from evil, placing oils on the belly, wearing amulets, ingesting invoked water, wearing a strand of invoked jute, and applying herbs to the stomach.

In addition, the study showed that a woman’s immediate family may influence her health behaviors during pregnancy. Some of the women we interviewed were part of a family system that insisted on following cultural rituals, whereas others rejected them. These families also held strong and divergent opinions about the ANC offered by clinical providers; in some instances, such divergent views could be seen within the same extended family network. Beyond the family, important leaders such as community elders, religious leaders, and market women (women who run small business enterprises in Sierra Leone and have influential roles in local economies in the country) contributed to a woman’s worldview drawn from both clinical and cultural viewpoints for understanding pregnancy and related health behaviors. We define that worldview as a healthworld.^14^

Participants’ responses organized under this topic fell into four categories: clinical/medical healthworld, spiritual/traditional healthworld, a hybrid healthworld relying on both clinical/medical and spiritual/traditional understandings, and a healthworld marked by tension between clinical/medical and spiritual/traditional understandings.

#### Clinical/medical healthworld.

Forty-seven out of 399 responses by focus group participants or interviewees (11.8%) were coded as a “clinical/medical healthworld.” The most common clinical/medical healthworld response came when participants were asked about the best location for delivery. Twenty-three participants named a hospital or health center, because complications could be easily managed there. Such responses came from mothers, pregnant women, husbands, clinical providers, traditional health practitioners, and religious leaders. A clinical/medical healthworld was also reflected in participants’ responses to the importance of ANC, which stressed the importance of early, ongoing ANC visits. Although this perspective was shared by all clinical providers interviewed and most traditional health care providers, it was not universally shared among members of the community. A noteworthy finding is that the importance of ANC visits was quite high among women who had experienced complications during earlier pregnancies and in women who had experienced a miscarriage or stillbirth.

#### Spiritual/traditional healthworld.

More responses, 165 out of 399 (41.4%), were coded to reflect a spiritual/traditional healthworld than a clinical/medical healthworld (*N* = 47, or 11.8%). The topics coded as spiritual/traditional healthworld reflected the influence of traditional beliefs on pregnant women’s health behaviors, how gendered social norms set by spiritual/traditional beliefs (including global religious traditions such as Christianity and Islam) affect women’s options for sexual and reproductive health services, the greater trust afforded to traditional health practitioners over clinical providers and the greater capacity of spiritual/traditional beliefs and practices to explain and treat complications in comparison with clinical care.

Participants described numerous ways in which a spiritual/traditional healthworld influences pregnant women’s health behaviors. A clinical provider from Sierra Leone noted the contradictions between spiritual beliefs and the doctrines of Christianity and Islam. Participants from different countries described women’s decisions to delay ANC until their pregnancies were physically noticeable for fear that some dangerous person in their community might use a spiritual practice to cause harm to the fetus (for example, casting a spell or invoking an evil eye). They noted that a fetus who survived into the second trimester was less vulnerable because it was more firmly implanted in the womb.

A spiritual/traditional healthworld also contributed to norms that influenced women’s options for sexual and reproductive health services. A clinical provider in Kenya remarked that the greater value placed on male babies leads to large families in parents’ hopes of having more male children. They also described how this norm sometimes led to attempts at an elective abortion, a procedure that is illegal in Kenya, or encouraged gender-based violence when a male spouse blamed the mother for giving birth to a girl. Another provider in Kenya noted that the timing of ANC visits was often determined not by women but by their husbands. A provider in Ethiopia noted that clinic policies dictate that a husband consent to any treatment of complications that arise during the pregnancy or present during delivery.

Some participants described the superiority of a spiritual/traditional healthworld in comparison with a clinical/medical one. Traditional health practitioners described the greater trust placed in them in comparison with clinical providers in their communities. A spiritual/traditional healthworld was valued by some for offering better explanations and treatments for complications. A traditional birth attendant in Kenya described “rariu,”^23^ a complication seen during pregnancy and understood among the Luo ethnic group as something clinical providers cannot diagnose or treat. A woman in Sierra Leone described the medicine that a Sowei, a traditional practitioner, can provide to stop a miscarriage when clinical providers are helpless. Women in Mozambique described two conditions—nhacua and umbua—that can only be treated by traditional health practitioners. A traditional provider in Sierra Leone noted that only traditional practices can protect a baby if the devil fights the mother for the child and warned that taking the child to the hospital in such instances will lead to the child’s death. An elder woman in Bangladesh described the multiple health benefits of spiritual practices and the widespread use of blessed items such as threads, water, and amulets. A Hindu religious leader there described the importance of the ritual of Shashti Puja to ensure a newborn child’s health and development. Multiple women who had experienced a miscarriage or stillbirth described the futility of clinical interventions because they believed God had willed the event to occur.

Finally, two participants from Kenya rejected traditional spiritual practices, not because they contradicted clinical practice, but because they were contrary to their own Christian beliefs. Although other participants described the integration of traditional beliefs and practices alongside their religious beliefs, this perspective echoes other ethnographic research in Kenya describing a rejection of traditional practice as a hallmark of faithful adherence to Christianity.^24^

#### Hybrid healthworld.

A hybrid healthworld employs both clinical/medical and spiritual/traditional understandings. Such perspectives were quite common; 123 responses of 399 total (30.8%) were coded to reflect a hybrid healthworld, with responses coming from traditional practitioners (92), women who have experienced a miscarriage (7), women who were pregnant or recently gave birth who previously had a child (6), clinical providers (5), mothers-in-law of pregnant women (4), women who experienced a stillbirth (3), parents of pregnant women (3), a community leader (1), a religious leader (1), and a woman experiencing her first pregnancy (1).

We focus our analysis of a hybrid healthworld on the relationship between clinical providers and traditional practitioners. A large body of literature details tensions between clinical and traditional providers, along with less frequent examples of collaboration.^25^^–^^31^ Participants in our study also described such tensions; however, we found more frequent descriptions of collaboration in our study, coming from 65 responses. Some of those we interviewed described the possibility of collaboration, but most described actual collaboration already occurring in all five countries. For example, a Christian religious leader from Mozambique described the importance of such collaboration between traditional birth attendants and clinical providers, especially for women in the community with few economic resources. Forty-two different responses (65%) prioritized clinical/medical care and framed spiritual/traditional practices as a complement. Only 11 responses—all from traditional health providers—valued both perspectives but prioritized the spiritual/traditional over the clinical.

#### Tension between healthworlds.

A description of tensions between a clinical/medical and a spiritual/traditional healthworld was less common than a hybrid description, with 64 of 399 (16.0%) responses coded as tension between healthworlds. Twenty-four responses (37.5%) reflected the perspective that a clinical/medical healthworld should take precedence in response to such tension. The influence of traditional herbs and medicines was the most frequently cited tension because clinicians noted incompatibilities between the medicines they dispensed and the medications dispensed by traditional practitioners and expressed concerns about the effects of drug-drug interactions. Four clinical providers claimed that clinical/medical healthworlds reflected actions based in science whereas spiritual/traditional practices were grounded in trial and error. Five participants representing both clinical providers and community members said that traditional/spiritual beliefs were dangerous, ineffective, and contradicted clinical practice. In contrast, eleven participants representing traditional practitioners and community members claimed that the spiritual/traditional took precedence when tensions arose and noted the higher level of trust in indigenous practices, whereas clinical medicine was less trusted because foreigners introduced it.

### Tension or alignment between community perspectives and clinical management of pregnancy.

In addition to the thematic analysis described above, we also examined responses to specific questions about ANC or labor and delivery asked during KIIs or FGDs. This additional analysis offered insight into participants’ direct perceptions of these two topics; 361 responses to such questions were first clustered into two subgroups: alignment or tension with ANC and FBD. Those excerpts were organized into eight categories that were articulated according to the ways in which alignment or tension was framed in the excerpt (Table 4).

**Table 4:** Cross-cutting topics reflecting alignment or tension with pregnancy-related services

Topics (A total of 361 excerpts coded for alignment or tension with ANC or facility delivery and organized along these eight categories)	Alignment (*n* = 259, 71.7%)		Tension (*n* = 102, 28.3%)	
	ANC (124)	Facility delivery (135)	ANC (68)	Facility delivery (34)
1. Attitude toward Clinical Care (*n* = 48)	16	18	10	4
2. Attitude toward Traditional Care (*n* = 68)	15	21	20	12
3. Clinical Resources/Services (*n* = 26)	10	12	2	2
4. Community Influence (*n* = 13)	4	8	0	1
5. Family Influence (*n* = 38)	10	22	2	4
6. Innovations (*n* = 4)	2	2	0	0
7. Motivation (*n* = 45)	26	4	15	0
8. Perceptions/Experiences (*n* = 119)	41	48	19	11

ANC = antenatal care.

Seven of the categories were cross-cutting, sometimes informing perspectives that aligned with ANC and FBD but informing perspectives in tension with these topics at other times. The category “innovations” only reflected alignment. Participants offered perspectives that aligned with ANC and FBD with far higher frequency (259 out of 361; 71.7%). Responses that aligned with FBD were slightly more frequent (135 of 259; 52.1%) than those that aligned with ANC, whereas responses in tension with ANC were almost twice as frequent (68 of 102; 66.7%) as those in tension with FBD.

#### Attitude toward clinical care.

Responses organized under the category “attitudes toward clinical care” were linked both to alignment and tension. Responses clustered under alignment with facility delivery included education on the importance of facility delivery and skilled management of complications during delivery, including delivery of twins, obstructed labor, fetal distress, and prolonged labor. The response clustered under tension reflected a perspective that clinicians’ decisions are random and do not address the needs of the mother or child. Most of the traditional providers we interviewed described their respect for and appreciation of clinical obstetric care, but two criticized FBD. Traditional providers who offered delivery services noted the need for referral to FBD sites if a complication arose and acknowledged the challenges involved with such referrals.

#### Attitudes toward traditional care.

Traditional beliefs and practices could either align with or challenge clinical ANC and FBD. Traditional practitioners we interviewed supported clinical ANC and saw their role as complementary. In addition, two clinical providers also described collaborating with a traditional practitioner. Seven traditional practitioners and two family members of pregnant women claimed that traditional practitioners could better manage certain pregnancy complications. Finally, two traditional providers supported clinical ANC in general but were critical of the care provided by certain facilities. Two pregnant women trusted traditional providers more than clinical providers, seeing them as an alternative to clinical ANC, not as a complement.

#### Clinical resources and services.

Responses categorized under clinical resources and clinical services were also linked to both alignment and tension. Two responses in tension with ANC described clinical services that did not align with cultural practices, whereas responses categorized by tension related to FBD referenced poorly equipped facilities and the lack of transport. Twelve participants described perceptions and attitudes that aligned with ANC, noting the value of laboratory services to inform decision-making, medications to manage complications, and ultrasounds to aid in diagnostic screening and care. Twelve participants who delivered in a facility appreciated the capacity to manage complications.

#### Community influence.

Participants described two community resources that encouraged uptake of ANC services: faith communities and schools. Friends were frequently named as an important resource in supporting women who experienced a miscarriage. Several community resources reflected alignment with FBD. These included neighbors, who were often named as a source of financial support; religious leaders, who held a distinctive authority in advocating for clinical care and facility deliveries; and religious communities, who also offered financial and emotional support. However, one religious leader also offered two perspectives in tension with FBD. First, the religious leader described negative perceptions toward women who were pregnant outside of marriage, noting that they keep their pregnancy secret and do not seek care. Second, the leader criticized pain management during delivery as futile, using the Bible to frame such pain as a consequence of sin (this was likely a reference to the biblical story in the book of Genesis 3:16).

#### Family influence.

The influences of family could reflect both tension and alignment with clinical management of pregnancy. Some participants described the importance of different family members in supporting their uptake of ANC. Various women described how their husbands insisted they attend ANC services instead of seeing a traditional provider. Others described the financial support of many different family members that made ANC visits possible. Family members who were also clinical providers were quite influential in participants’ perceptions about ANC services. At the same time, both the husband’s influence and family members’ financial circumstances were also named as tensions. One participant described her husband’s insistence that she follow pregnancy advice from an elder woman in the community instead of following that of clinical providers. Many participants described the stress of ANC and obstetric costs in circumstances where they and their extended family were in financial distress. Family members offered important support that aligned with facility-based labor delivery, including encouragement, financial support, emotional support through a cesarean section, and advocating for a mother during the labor and delivery process.

##### Innovations.

Participants described innovations to increase uptake of ANC and FBD. Suggested strategies to support expectant mothers in going to ANC visits included offering support for childcare, providing transportation, and enlisting the family system by offering incentives for the husband’s involvement or offering formal recognition to mothers and/or mothers-in-law who support women’s attendance. The partner of a pregnant woman in Mozambique mentioned the importance of partners being invited to accompany pregnant women to appointments. Women were often important advocates for other women. Many of the women we interviewed who had previously given birth described their efforts to encourage pregnant women to seek out early, ongoing ANC services and make a care plan for a facility delivery. They also encouraged women to set money aside to cover the costs of pregnancy and delivery. One clinical site in Bangladesh helped patients cover expenses from a savings plan that paid interest income on money set aside. Participants noted that this site did not demand prepayment but offered services regardless of ability to pay.

##### Motivations.

Participants described a variety of motivations for their pregnancy-related beliefs and practices. Two types of motivations reinforced alignment with ANC: intrinsic motivations in relation to health behaviors they perceived to encourage a healthy pregnancy and delivery and increased motivations due to pregnancy complications. These same types of motivation also contributed to tensions with ANC. Several women whose fetus was not in the correct position for easier delivery late in their pregnancy described how traditional providers worked to shift the fetus through touch and massage, whereas clinical providers did nothing. Some pregnant women described no intrinsic motivation for ANC, most often due to a perception that an ANC visit made the pregnancy public, leaving women and their fetuses vulnerable to harm from witchcraft. However, sometimes the lack of motivation was simply prioritizing other daily concerns over an ANC visit.

#### Experiences and perceptions.

For women who had experienced an earlier pregnancy, attitudes toward FBD were influenced by these previous experiences. Women whose experiences with a FBD were positive described clinical providers who were responsive and kind; they appreciated receiving care not provided by traditional birth attendants at home. Sometimes, earlier experiences reflected a tension with FBD. Some women blamed a complication during a previous pregnancy on the clinical care they had received and were suspicious of clinical providers. Others trusted traditional providers and refused to consider facility delivery.

## DISCUSSION

We conducted a multisite qualitative study to understand the influences of common perceptions, beliefs, and practices related to pregnancy and birth outcomes on 1) individual and community understandings of the causes of complicated or uncomplicated pregnancies and birth outcomes, and 2) attitudes toward antenatal, obstetric, and postpartum clinical care. Although this larger study led to several findings, this paper focuses only on those that help us understand social–cultural knowledge and beliefs about pregnancy, identify community members’ attitudes and perceptions of pregnancy-related clinical care, and describe how women’s health-seeking behaviors are informed by such beliefs and perceptions. These topics are important to efforts across the CHAMPS network to increase the uptake of ANC and FBD.

Having described the influences of both clinical and cultural frameworks for understanding pregnancy and providing specific examples of alignment or tension between community members’ beliefs and ANC or FBD, we turn now to two challenges hindering uptake of pregnancy-related clinical care and two recommendations that could increase uptake of such care. The challenges include: 1) suspicion of traditional practitioners by clinical providers and 2) cultural beliefs at odds with uptake of ANC, especially in the first trimester. Finally, we offer two recommendations: 1) identifying trusted partners who could foster collaboration between clinical and traditional providers and 2) encouraging the expansion of low-cost support services.

### Challenge: Suspicion of traditional practitioners by clinical providers.

We found a high level of suspicion of traditional providers by clinical providers. Such suspicion was seen in the healthworlds theme and in the alignment/tension assessment. Clinical providers described tensions between the clinical/medical and spiritual/traditional healthworld more frequently and were less likely to describe a hybrid healthworld in comparison with traditional providers or members of the community. There were a few descriptions of collaboration between clinical and traditional practitioners, particularly in Sierra Leone, which has a history of collaboration between the Ministry of Health, Christian religious leaders, Muslim religious leaders, and traditional health practitioners in response to health emergencies.^32^ This suspicion negatively influences referrals from traditional providers to clinical sites and may force women and families to choose one type of health services over another, even though they see the value of both.

### Challenge: Cultural beliefs at odds with ANC, especially in the first trimester.

There are strong, well-established cultural beliefs and related practices that present obstacles to increasing uptake of ANC in the first trimester. Many women who wait to present for care understand pregnancy risks through a spiritual/traditional healthworld. Across all five country sites, participants offered descriptions of numerous spiritual dangers in announcing a pregnancy too early that leaves pregnant women and their fetus at risk. These include witchcraft, the evil eye, evil charms, evil air flow, and malevolent spirits. Such descriptions offered in our qualitative study are reinforced in other studies.^33^^–^^36^

### Recommendation: Identifying trusted partners to foster collaboration.

Although spiritual beliefs may challenge early uptake of ANC, they can also provide a rationale for uptake of ANC and FBD. Numerous community leaders championed a hybrid healthworld, and the responses categorized as aligning with ANC or facility-based care offered numerous descriptions of such a healthworld that values both clinical care and traditional practices. Partnerships between clinical providers, traditional practitioners, and religious leaders could foster such uptake. Because many of these trusted messengers frame their support from a spiritual/traditional frame, their advocacy messages are received differently than those of clinical providers. For example, a few studies indicate that some Muslim women present for early ANC because of teachings about the concept of ensoulment,^37^^,^^38^ even when an appeal to the clinical benefits of early ANC is unpersuasive. Although our study did not specifically ask about ensoulment, a clinical provider from Ethiopia alluded to it. Ensoulment describes the point, generally assumed to occur at approximately 120 days, when Muslims believe that the soul enters the fetus and bestows on it the same moral significance as a human being. This teaching leads some Muslim faithful to screen the fetus for developmental abnormalities early in the first trimester because elective abortion procedures are strictly forbidden after ensoulment occurs, except in rare cases.^39^^,^^40^ Given the pervasive influence of spiritual beliefs on pregnancy beliefs and behaviors and the high levels of trust placed in traditional providers by many participants suspicious of ANC and FBD, messaging and other types of collaboration between clinical providers and trusted community partners could change perceptions about ANC and facility deliveries.

In addition, women who have experienced a miscarriage or stillbirth can be trusted messengers by drawing on their own experiences. In our qualitative study, these women were far more likely to support early, regular ANC and facility delivery than other women we interviewed because of the loss they had suffered, and they described their own attempts to champion early ANC and facility delivery. These findings are echoed in the literature.^41^^–^^45^

### Recommendation: Encouraging the expansion of low-cost support services.

When asked to identify the kinds of services that would be most helpful for clinical facilities to provide pregnant women and their families, few participants named highly technical or complicated clinical services. Rather, they described the difference that simple resources such as diapers, food support, a wash basin, and a hat could make. When asked to identify the reasons for negative perceptions about clinical services, participants described rude, disrespectful treatment and clinical staff who kept them waiting or canceled appointments more often than they did the poor quality of clinical care.

Clinical care must be both technically sound and respectful. Actions to improve perceptions by only focusing on low-cost responses without addressing the level and quality of care would ignore principles of public health action based on evidence. Nonetheless, efforts to offer the resources that participants value could build support for clinical care by demonstrating that patients’ perspectives matter. Because the suggestions are far less expensive than many complex clinical services, they could be implemented even as efforts to strengthen clinical care capacity continue.

### Limitations of the study.

The participation of multiple different sites in Africa and a site in South Asia is a strength of this study, providing ideas that may be useful for health leaders in those regions to consider as they work to bring more pregnant women into care. Because of the methodological challenges in definitively determining the meanings of subjective perceptions across diverse and disparate cultural contexts, this study was not intended to generate generalizable conclusions. Rather, its purpose was to provide complex descriptions of the cultural factors that influence the beliefs of women and their families related to pregnancy and the health services that support the delivery of healthy infants. Knowledge of these perceptions has been used to inform pregnancy surveillance efforts at CHAMPS sites as well as data-to-action initiatives across the CHAMPS network that use epidemiological and clinical data generated by CHAMPS to inform public health action for improved maternal and child health.

## CONCLUSIONS

Low uptake of pregnancy-related clinical services contributes to high rates of maternal and neonatal deaths. However, efforts to increase the uptake of clinical services that ignore cultural beliefs are unlikely to succeed. Understanding cultural beliefs about pregnancy and the ways in which those beliefs influence health behaviors and decision-making of pregnant women will help address the barriers to uptake and improve the quality of care. Such efforts must also consider how community members’ experiences in clinical settings and with traditional health practitioners influence their attitudes and perceptions about ANC and FBD. This study has described such beliefs, practices, attitudes, and perceptions to inform more effective public health action.

## Supplemental Materials

10.4269/ajtmh.26-0519 Supplemental Materials
