# Supplementary material for: Beliefs About Pregnancy and Perceptions of Clinical Care Influencing the Uptake of Antenatal Care and Facility-Based Delivery: Findings Across the CHAMPS Network
Source: Am J Trop Med Hyg. 2026 Nov 10:tpmd260519. Online ahead of print. doi: 10.4269/ajtmh.26-0519 (PMC13770351; doi:10.4269/ajtmh.26-0519)
Supplement: Supplementary file 1 [file tpmd260519.SD1.pdf]

This supplementary material contains excerpts from transcripts of interviews and focus group discussions that inform the topics explored in the findings of the research described in the manuscript. The link below takes the reader to an Excel document with the excerpts organized as follows:

1) The three sheets in the Excel file correspond to the three topics explored in the findings:
    a. clinical factors
    b. healthworld
    c. perspectives reflecting alignment or tension with antenatal care (ANC) and/or facility-based delivery.
2) Each entry in the sheets for "clinical factors" and "healthworld" is listed by row. Each row is then organized by columns.
    a. Columns A-D list the main theme and any sub-themes that further describe how the excerpt relates to the main theme.
    b. Column E identifies the country in which the interview or focus group discussion took place.
    c. Column F identifies the type of community member interviewed according to the sampling frame described in the manuscript.
    d. Column G contains the excerpted text from the transcript of the interview or focus group discussion.
3) Each entry in the third sheet, alignment or tension with ANC and/or facility-based delivery, is also listed by row with each row organized by columns. However, the columns reflect a different analysis and organization of the data. We analyzed any reference to ANC or facility-based delivery regardless of how the narrative was coded and assessed whether the perspective offered by the participant(s) in that text reflected alignment or tension with to ANC or facility-based delivery.
    a. Column A indicates whether the identified excerpt referenced ANC or facility-based delivery.
    b. Column B indicates whether the excerpts reflected tension or alignment
    c. Column C-E described the topic(s) within the excerpt relative to alignment or tension. All entries were coded with at least one topic (column C) with further elaboration on topics for columns D and E.
    d. Column F identifies the country in which the interview or focus group discussion took place.
    e. Column G identifies the type of community member interviewed according to the sampling frame described in the manuscript.
    f. Column H contains the excerpted text from the transcript of the interview or focus group discussion.

All entries were screened to remove information that could potentially identify the person being interviewed, a member of the community that the person being interviewed was describing, or a facility in which either clinical or traditional health services are offered. In such instances, redacted text appears in the excerpt with XXXXX replacing the original text.

The Excel file can be accessed at this link: https://docs.google.com/spreadsheets/d/11fcLKl-wHUbDShHllPN9sNMD5EQdSTm1/edit?usp=sharing&ouid=106420486567400161416&rtpof=true&sd=true
